# Direct Methods Optimised for Solving Crystal Structure by Powder Diffraction Data: Limits, Strategies, and Prospects

**DOI:** 10.6028/jres.109.009

**Published:** 2004-02-01

**Authors:** Angela Altomare, Carmelo Giacovazzo, Anna Grazia Giuseppina Moliterni, Rosanna Rizzi

**Affiliations:** IC-CNR c/o Dipartimento Geomineralogico, Università di Bari, Campus Universitario, Via Orabona 4, 70125 Bari, Italy

**Keywords:** direct methods, intensity extraction, structure solution by powder data

## Abstract

The *ab-initio* crystal structure solution by powder diffraction data requires great efforts because of the collapse of the experimental information onto the one dimensional 2*θ* axis of the pattern. Different strategies will be described aiming at improving the process of extraction of the integrated intensities from the experimental pattern in order to make more straightforward the structure solution process by direct methods. Particular attention will be devoted to the EXPO program. Some of its performance will be analysed and results will be shown.

## 1. Introduction

Two approaches can be followed for solving *ab-initio* crystal structures by powder diffraction data: *the traditional approach* and the *direct space approach*. Both of them require the knowledge of the following minimal information: a) experimental diffraction profile; b) cell parameters; c) space group; d) unit cell content. In addition, the direct space methods (simulated annealing, genetic algorithm, Monte Carlo techniques) need the knowledge of the structure molecular geometry also. When this information is not available, the traditional method is the obligatory choice.

The main steps of the traditional approach consist of: 1) indexing the powder pattern; 2) determining the space group; 3) solving the structure by direct methods, Patterson methods or Maximum Entropy; 4) refining the structure by the Rietveld method.

About point 3), direct methods are based on statistical and probabilistic calculations. They use the experimental |***F_h_***| structure factor modulus corresponding to each ***h*** reflection (see Ref. [2] for direct methods theory) and aim at solving the phase problem. The inverse Fourier transform of the ***F_h_*** structure factors provides the electron density map whose maxima correspond to the atomic positions. Direct methods are successfully applied to single crystal data. In case of powder solution, the extraction of the integrated intensity ***I_h_*** (***I_h_*** ∝ |***F_h_***|^2^) for each reflection from the experimental pattern is preliminary to the application of direct methods. It requires the decomposition of the experimental profile into the single peaks and the area under each peak gives the wanted ***I_h_*** value. Unfortunately, some problems occur and make the extraction procedure very critical for the success of the structure solution step. The main problems regard:

- the peak overlap. It depends on the experimental resolution and on the structure complexity. It increases at large 2*θ* angular values of the observed pattern;
- the background. Its estimate is not trivial. It adds to the peak overlap effect so that, especially at large 2*θ* values, it is difficult to estimate the noise contribution correctly;
- the preferred orientation. The crystallities are not always randomly oriented. This behaviour modifies the ratios of the experimental intensities.

For the above mentioned problems the |***F_h_***| estimate process reveals itself as a crucial point in the powder *ab-initio* solution: the more reliable the extracted integrated intensities values, the larger the success probability of solving the structure.

## 2. The Integrated Intensity Extraction Process

Two methods are widely used for extracting the integrated intensities from the powder pattern: the Pawley method [3] and the Le Bail method [4].

The Pawley method is based on a non linear least squares procedure. The integrated intensities are refinable variables in addition to the profile parameters. Because of the peak overlap, the least squares are often unstable and they provide negative integrated intensity values which must be discarded. For this reason the method needs positivity constraints [5], [6].

The Le Bail method is an iterative decomposition algorithm following the Rietveld formula [7]. The integrated intensity value is calculated according to:

$$I_{h}=\sum_{i}{\left(y_{\text{obs}}(i)-y_{b}(i)\right)}^{*}\;y_{\text{calc}}(i,h)/\sum_{k}y_{\text{calc}}(i,k).$$

where the summation is over the peak range, *y*_obs_(*i*) is the experimental count in the 2*θ_i_* angular value, *y*_b_(*i*) is the background contribution, *y*_calc_(*i*, ***h***) is the calculated count in 2*θ_i_*, due to the ***h*** reflection contribution. The Le Bail method starts with arbitrary but fixed integrated intensity values and the formula is cyclically applied. It is rapidly convergent; it provides positive values if the background is properly estimated, but it tends to equiportion the intensity of a group of reflections strongly overlapping.

## 3. The Direct Methods Efficiency With Powder Data

In order to assess which of the two above mentioned methods is more suitable to be combined with direct methods, it proves useful to take into account the reliability parameter *R_F_* about the extracted amplitudes:

$$R_{F}=\frac{\sum \left|{\left|F\right|}_{\text{true}}-{\left|F\right|}_{\text{extracted}}\right|}{\sum {\left|F\right|}_{\text{true}}}$$

where the summation is over the number of reflections, |***F_h_***|_extracted_ is the structure factor modulus extracted by one of the two methods and |***F_h_***|_true_ is the structure factor modulus calculated by using the published atomic parameters. The reliability parameter *R*_p_ about the profile:

$$R_{p}=\frac{\sum \left|\left|y_{\text{obs}}(i)\right|-\left|y_{\text{calc}}(i)\right|\right|}{\sum \left|y_{\text{obs}}(i)\right|}$$

is considered also. The summation is extended to the number of profile counts, *y*_obs_(*i*) and *y*_calc_(*i*) are the observed and the calculated counts, respectively.

In Table 1, crystal chemical information are given for some test structures (the code name, the space group, the unit cell content, the 2*θ* experimental range and the number of reflections in the range). They cover a quite large variety of cases.

In Table 2, for each of some test structures, the *R*_F_ and the *R*_P_ values are shown. They are calculated by using the integrated intensities extracted by the EXTRA program [8] and the ALLHKL program [3], respectively. EXTRA is a Le Bail based package, ALLHKL uses the Pawley method. In Table 2 the *R*_p_ values are small but the *R*_F_ values are large (0.4 is the average value). This means that: a) low *R*_P_ value is necessary and not sufficient condition for a reliable extraction; b) the integrated intensity accuracy is very low and this is the reason for which the powder *ab-initio* solution is not straightforward. Moreover, the *R*_F_ values by EXTRA are always smaller than the *R*_F_ values by ALLHKL so that we could conclude that the Le Bail method should be preferred but that behaviour may depend on the equipartition tendency of the Le Bail approach. However we proved that the statistical efficiency of direct methods improved by using the Le Bail extracted intensities [9].

## 4. The Le Bail Method Advantage

The Le Bail method offers a great advantage: it is very sensitive to the starting point. This aspect is shown in Table 3 where, for some test structures, the *R*_F_ values are shown. Protocol 1 corresponds to the traditional Le Bail and Pawley extraction cases respectively, while Protocol 2 corresponds to the case when the true integrated intensities are used as starting values in the Le Bail and Pawley methods, respectively. The values in Table 3 suggest that the integrated intensity estimate is not sensitive to the starting point if the Pawley method is adopted. On the contrary, it is not so for the Le Bail approach. This means that if the starting integrated intensities are less arbitrary and closer to the true ones the amplitude estimate is improved. On the other hand, the values of Protocol 2 represent the maximal accuracy level we can reach. The great advantage preserved by the Le Bail method can be exploited. From this last consideration the EXPO program was developed [1].

## 5. The EXPO Program

**Fig. 1:**
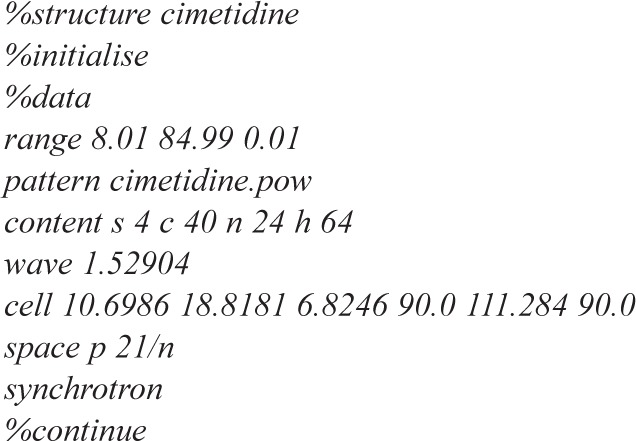
EXPO minimal input: an example.

EXPO is the integration of EXTRA and SIR-POW[10] programs. This last is devoted to the structure solution by direct methods. EXPO needs the minimal information about the experimental powder pattern, the cell parameters, the space group and the unit cell content (Fig. 1 shows an example of the minimal EXPO input). Its main steps are:

1. Extraction of the integrated intensities (EXTRACTION routine);
2. Normalization of the extracted intensities (NORMALIZATION routine);
  The normalization rule restrains that:
  $$<{\left|E_{h}\right|}^{2}>\;=1$$
  where ***E_h_*** is the normalized structure factor. The large |*E*| value reflections are statistically meaningful. The statistical analysis of the normalized structure factors can reveal the presence of pseudo-translational symmetry and/or preferred orientation.
3. Calculation of the structure invariant relationships (triplets and quartets) (INVARIANT routine);
  The structure invariant statistical reliability is taken into account.
4. Phasing the reflections (PHASE routine);
  Random phases are given to few pivotal reflections and the phase information is expanded to the large |*E*| value reflections by using the most reliable triplets. The phasing trial corresponding to the largest combined figure of merit (CFOM) is selected.
5. Calculation of the Fourier map (FOURIER routine). The selected phases are used for calculating the Fourier map whose maxima are searched and chemically interpreted. The map is optimized by combining successive structure factor calculations with preliminary least squares cycles.

Therefore, EXPO is a program able to reach the structure solution starting from minimal experimental information. Thanks to the Le Bail tendency to be very sensitive to the starting point, EXPO is more than the trivial combination of the two programs. It is able to exploit information becoming available during the structure solution process itself in the extraction routine to improve the structure factor modulus estimate.

## 6. The Use of Prior Information

The following types of information provided by the solution process can be used as prior information for improving the extraction of the integrated intensities:

- Pseudo-translational symmetry information [11].
  When a structure is affected by pseudo translational symmetry, a percentage of its electron density repeats itself after an u vector shift. This means that
  $$\rho_{p}(r)=\rho (r+u)$$
  where *ρ_p_* is a *p* percentage of the electron density and *u* is the pseudo-symmetry vector. In EXPO, the statistical |*E*| value analysis is able to reveal the presence of pseudo-symmetry, to recognize the percentage (the FSP fractional scattering power) and the type (the *u* vector). If this pseudo-symmetry occurs, an *α**_h_*** coefficient is associated to each ***h*** reflection so that, if *α**_h_*** is equal to zero, the reflection is said to be a superstructure reflection, on the contrary it is a substructure reflection. In the pseudo-symmetry case, the normalization rule is violated and
  $$<{\left|E_{h}\right|}^{2}>\;=1+\left(\alpha_{h}-1\right)\cdot FSP.$$
  This statistical information can be exploited in a successive intensity-recycled Le Bail extraction. In this case, the starting casual integrated intensities are modulated by the statistical term in the previous formula [1 + (*α**_h_*** − 1)·*FSP*]. So doing, the substructure reflection intensities are increased and the superstructure reflection intensities are decreased. The new intensity estimates are more accurate than the traditional Le Bail extraction ones and the phasing process gives better results.
- Probabilistic estimate information [12].
  In the INVARIANT routine, EXPO is able to provide the probabilistic estimate of the structure factor modulus (the positivity condition of the electron density is considered in the reciprocal space) by using triplet relationships both in the centric case and in the acentric case. A intensity-recycled Le Bail extraction can be carried out by exploiting the amplitude statistical estimates as starting values.
- The Patterson information [13].
  EXPO is able to calculate a Patterson map by using the extracted integrated intensities from a traditional Le Bail extraction. The map is modified (the origin peak is reduced and the low intensity points are put to zero). After that, the map is inverted. The thus obtained squared structure factor moduli can be exploited as starting point in a new Le Bail extraction.
- The located fragment information [14].
  If a traditional Le Bail extraction EXPO run is able to locate a fragment in correct way the structure factor moduli calculated by taking into account the recognised atomic positions can be used as starting point in a intensity-recycled Le Bail process.

We can summarise that the Le Bail potential to be sensitive to the starting point can be exploited by considering different kinds of prior information to make the starting point closer to the true one. In this way, the extraction is more efficient and the structure solution results become more reliable. In Table 4, the results concerning the use of prior information are shown. The *R*_F_ value corresponding to the traditional Le Bail extraction run (*R*_D_) and to the use of pseudo-symmetry information (*R*_PSEUD_), Patterson information (*R*_PATT_) and probabilistic estimate (*R*_PROB_) are given. The last column corresponds to the use of the true intensities to start the Le Bail algorithm. The results in that table show that the use of prior information decreases the *R*_F_ values respect to the traditional Le Bail extraction case, making them closer to the values in the last column. The pseudo-symmetry information can be applied if it is revealed. In Table 5, the *R*_F_ values obtained by using the fragment information (*R*_FRAG_) with the traditional Le Bail extraction *R*_D_ value and the selected fragment in the asymmetric unit (in parentheses the corresponding percentage) are given for some test structures, confirming the advantage in exploiting prior information. Therefore, the use of prior information can help when the obtained traditional Le Bail extraction solution is not reliable. The following suggestions can be taken in consideration for optimise its use and for avoiding the bad combined use of prior information because of their correlation:

1. if pseudo-symmetry is revealed, and especially when the detected percentage is large, it is convenient to use it;
2. if no pseudo-symmetry effect is detected, but the structure contains heavy atoms, the use of Patterson information can improve the results;
3. the probabilistic estimate information can be used in all the cases;
4. if a fragment is located it can be exploited.

The structure solutions supplied by EXPO are shown in Table 6, where for each test structure we have: the maximum (sin*θ*/*λ*)^2^ value, the number of reflections, the corresponding number of independent observations (see [15] for details), the number of atoms to find in the asymmetric unit and the number of atoms found by EXPO (in a traditional Le Bail extraction or intensity-recycled run). Most of the structures are completely solved. This doesn't occur when the data quality is poor (small (sin*θ*/*λ*)^2^ value and/or a large overlapping degree).

## 7. The Random Approach

When no prior information is available, or when it is poor, a recently developed procedure can be attempted [16]. It is based on a random approach and it works so that, for each cluster of overlapping reflections, some random partitions of the cluster overall intensity are considered. The partition corresponding to the best fit (the lowest *R*_P_ value) in the cluster local range is selected as the most reliable one and it provides the integrated intensity values to use as starting ones in the Le Bail formula. The random procedure is applied before each Le Bail cycle. The merit of the new approach is to break the Le Bail tendency to equipartition the intensity of a group of overlapping reflections. Its aim is to modify the equipartitioned intensities: a necessary goal, if the modified intensities correspond to the pivotal reflections in the phasing process. The results of the random procedure are shown in Table 7, where the phase error in the traditional Le Bail extraction case (ERR1) and in the random case (ERR2) are given for some test structures. The values corresponding to ERR2 are always much better than ERR1. This means that the power of the new procedure, to modify a small number of reflections that are very important in the phasing process, remarkably improves the phasing process, even though, on average, no more accurate structure factor moduli estimates are obtained.

## 8. The POLPO Procedure

The solution provided by direct methods is frequently incomplete. In particular, this happens in the case of heavy atom structure when the heavy atoms are easily located, but the light atoms are hardly recognised. The traditional approach for completing a partial solution consists of combining Fourier map calculations with Rietveld refinement. The trend is not trivial, not automatic, not fast. The new POLPO procedure [17] has been introduced in EXPO for completing the structure when the structure cations are located. The procedure uses the polyhedral information and it is based on the Monte Carlo technique. The starting point is the cation positions supplied by direct methods. The user gives the polyhedral information by using directives about the polyhedron type, the corresponding cation label, the expected polyhedral average distance, the distance tolerance and the angle tolerance. The procedure automatically calculates the cation connectivity [17]. Several configurations obeying the requested polyhedral and connectivity rules are built. The geometrical construction takes into account the tolerance about the distances and angles. Some configurations are rejected because they are chemically inconsistent. Among the remaining possible configurations, the model corresponding to the best fit between the observed and the calculated profile (the lowest *R*_P_ value) is selected. Table 8 shows the POLPO results: the number of feasible obtained solutions, the lowest *R*_P_ value corresponding to the chosen model, the number of anions located in the asymmetric unit (in parentheses the true number), the average distances between the POLPO positions and the true ones and the CPU time are given. It can be seen that all the structures are completed in few time. The discrepancy with regard to the number of anions depends on the imperfectly located positions of the starting cations and on the fact that the construction by POLPO is carried out in a geometrically perfect way. The POLPO procedure is currently being enhanced with the aim of completing a structure when only some cations are positioned.

## 9. Conclusions

Thank to its graphical interface, EXPO is a very *user-friendly* program. It is able to give different opportunities for overcoming the difficulties in solving *ab-initio* crystal structures by powder diffraction data. The next version of EXPO will include N-TREOR [18], a modified and updated version of the program for indexing TREOR90 [19], the POLPO procedure and new strategies for optimising the Fourier map.

## Figures and Tables

**Table 1:** Code and crystal chemical information for the test structures. X: home diffractometer data; N: neutron data; S: synchrotron data

Code	Space Group	Unit cell content	2*ϑ* Range	Nr. reflection
AGPZ (X)	*Pbca*	Ag_8_N_16_C_24_H_24_	5.0–80.0	258
AND1 (S)	*P* 2_1_/*n*	C_28_N_20_O_8_H_44_	4.0–50.0	896
BACO (N)	*C* 2/*m*	Ba_4_C_8_O_20_D_8_	20.7–150.0	272
BAMO (X)	*P* 2_1_	Ba_4_Mo_12_O_40_	10.0–119.0	1220
BENZ (S)	*P*2_1_/*a*	C_24_F_12_	5.0–100.0	716
CF3BR (N)	*P*2_1_/*a*	C_4_Br_4_F_12_	6.0–150.0	375
CFCL (N)	*Fdd*2	C_8_F_16_Cl_16_	5.0–150.0	203
CFI (N)	*Cmca*	C_8_F_24_I_8_	10.0–150.0	428
CIME (S)	*P* 2_1_/*n*	S_4_C_40_N_24_H_64_	8.01–84.99	924
CROX (X)	$P\bar{1}$	Cr_8_O_21_	6.0–80.0	657
CUPZ (X)	*Pbca*	Cu_8_N_16_C_24_H_24_	5.0–80.0	243
DADA (X)	*P* 2_1_ 2_1_ 2_1_	Ti_8_K_4_Si_12_O_40_	10.0–95.0	518
EMT (S)	*P* 6_3_/*m m c*	(Si,Al)_96_Na_28_O_204_	4.5–63.0	670
GAPO (S)	*P b c a*	Ga_32_P_32_O_128_F_8_C_56_	7.0–63.69	1235
LAMO (X)	*P*2_1_/*a*	La_4_Mo_20_O_32_	11.0–69.0	271
LASI (N)	*P*2_1_/*c*	La_8_Si_8_O_28_	10.–115.72	253
LEV (S)	*R*3*m*	[Si_54_O_108_]3C_8_NH_16_	8.0–85.6	323
MCM (S)	*P* 6/*m m m*	Si_72_O_144_	2.2–50.0	480
MES (X)	*P*2_1_/*c*	C_24_N_4_O_20_S_4_H_52_	5.0–88.0	719
METYL (S)	I222	Na_16_C_16_H_48_	5.2–70.0	318
NBPO (S)	*C*2/*c*	Nb_20_O_120_P_28_	3.0–60.0	1201
NIZR (S)	*P*2_1_/*n*	Ni_4_Zr_8_P_4_O_16_	8.0–52.0	627
PBS (S)	*Pbca*	Pb_8_S_16_O_24_	7.5–79.8	477
SAPO (S)	*Pmmn*	Si_32_O_64_N_2_C_48_	5.0–79.98	716
SBPO (S)	*P*2_1_/*n*	Sb8 P14O48	6.0–100.0	1071
SGT (S)	*I* 4_1_/*a m d*	Si_64_O_128_C_104_	8.5–92.96	451
UTM1 (S)	*C* 2/*m*	Si_44_O_88_	2.5–49.97	1133
VFI (S)	*P* 6_3_	Al_18_P_18_O_114_	5.0–90.0	787
VNI (S)	*P* 4_2_2_1_2	Rb_44_K_4_Si_96_Zn_24_O_288_	5.0–60.0	1345
YONO (S)	*P* 2_1_	Y_8_O_26_N_2_H_18_	7.0–80.0	680
YURI (X)	*P* 2_1_/*c*	Na_4_S_4_O_16_C_12_F_2_	8.0–63.96	243

**Table 2:** The *R*_P_ and the *R*_F_ values corresponding to the intensities extracted by EXTRA and ALLHKL respectively

Code	EXTRA (Le Bail based)		ALLHKL (Pawley based)	
	*R*_F_	*R*_p_	*R*_F_	*R*_p_
AGPZ	0.53	0.12	0.60	0.15
BACO	0.35	0.05	0.39	0.05
BENZ	0.43	0.15	0.81	0.22
CF3BR	0.33	0.09	0.41	0.12
CFCL	0.22	0.06	0.34	0.06
CFI	0.51	0.03	0.78	0.05
CROX	0.39	0.09	0.60	0.13
CUPZ	0.50	0.06	0.86	0.11
LAMO	0.39	0.21	0.46	0.23
LASI	0.40	0.11	0.47	0.11
LEV	0.60	0.05		
MES	0.50	0.07	0.77	0.06
METYL	0.33	0.10	0.53	0.10
NBPO	0.43	0.10	0.52	0.18
NIZR	0.41	0.18	0.61	0.18
PBS	0.43	0.10	0.48	0.09
SAPO	0.47	0.06	0.87	0.12
SBPO	0.51	0.08	0.82	0.10
SULPH	0.35	0.03	0.47	0.08
YONO	0.33	0.10	0.44	0.10

**Table 3:** For each test structure: a) the *R*_F_ value by EXTRA and ALLHKL in a traditional extraction run (Protocol 1); b) the *R*_F_ value by EXTRA and ALLHKL by using the true (calculated by the published positions) |*F* | values as starting point (Protocol 2) are shown

Code	EXTRA		ALLHKL	
	Protocol 1	Protocol 2	Protocol 1	Protocol 2
AGPZ	0.53	0.26	0.62	0.61
BACO	0.34	0.21	0.39	0.39
BENZ	0.43	0.22	0.52	0.80
CF3BR	0.33	0.15	0.59	0.46
CFCL	0.22	0.12	0.34	0.27
CFI	0.50	0.32	0.74	0.84
CROX	0.39	0.16	0.55	0.60
CUPZ	0.50	0.24	0.86	0.74
LAMO	0.39	0.22	0.39	0.45
LASI	0.39	0.16	0.45	0.47
LEV	0.60	0.23		
MES	0.49	0.26	0.73	0.77
METYL	0.32	0.25	0.37	0.54
NBPO	0.44	0.12	0.52	1.11
NIZR	0.43	0.24	0.61	0.55
PBS	0.43	0.26	0.48	0.43
SAPO	0.47	0.19	0.87	0.86
SBPO	0.51	0.15	0.82	0.79
SULPH	0.34	0.20	0.44	0.56
YONO	0.31	0.18	0.32	0.42

**Table 4:** *R*_F_ reliability parameters (×100). *R*_D_ is in traditional Le Bail extraction case; *R*_PSEUD_ is in the prior pseudo-symmetry information case; *R*_PATT_ is in the prior Patterson information case; *R*_PROB_ is in the prior probabilistic estimate information case, *R*_TRUE_ when the true structure factor moduli are used

Code	*R*_D_	*R*_PSEUD_	*R*_PATT_	*R*_PROB_	*R*_TRUE_
AGPZ	52.28	33.51	38.93	47.05	24.18
BACO	31.32		28.09	28.22	16.94
BENZ	41.42		35.83	36.81	21.28
CF3BR	29.89		27.55	27.43	10.95
CFCL	21.14		15.64	19.29	9.99
CFI	49.29		45.04	46.23	30.25
CROX	36.65		33.11	31.06	15.71
CUPZ	47.07	34.79	34.49	41.21	21.90
LAMO	35.59		34.99	35.11	25.93
LASI	37.81		37.07	35.68	12.99
LEV	58.60		51.70	55.70	22.38
MES	46.39		44.08	42.09	25.65
METYL	28.98		27.13	27.31	22.74
NBPO	38.95	32.73	24.36	29.85	8.27
NIZR	42.17	41.65	37.07	36.06	21.25
PBS	40.82		38.10	38.32	26.50
SAPO	45.33		41.09	41.77	17.37
SBPO	48.56	30.85	28.88	31.69	13.17
SULPH	32.53		27.42	30.12	19.31
YONO	31.95		27.42	25.54	16.75

**Table 5:** For some test structures: the selected fragment and the corresponding percentage, the traditional Le Bail extraction *R*_F_ value (*R*_D_) (×100) and the *R*_F_ (×100) value when the fragment prior information is used (*R*_FRAG_) are given

Code	Selected fragment (%)	*R*_D_	*R*_FRAG_
AGPZ	1 Ag (97.2 %)	51.12	29.47
BAMO	2 Ba (37.8 %)	42.35	38.06
CUPZ	1 Cu (92.3 %)	46.98	26.53
DADA	1 Ti 2 K (54.4 %)	33.65	31.92
LAMO	1 La 2 Mo (55.8 %)	35.14	32.75
LASI	2 La (34.1 %)	37.68	33.55
NBPO	3 Nb (82.7 %)	40.19	23.99
NIZR	2 Zr (68.6 %)	41.85	36.09
SBPO	2 Sb (87.9 %)	49.52	23.25
YONO	4 Y (95.8 %)	31.93	20.15

**Table 6:** For each test structure: the maximum (sin*θ*/*λ*)^2^, the number of reflections, the number of independent observations, the number of atoms to locate (NATS1) and the number of atoms correctly located by EXPO in the asymmetric unit (NATS2) are given

Code	(sin*θ*/*λ*)^2^	*M*	*M*_ind_	NATS1	NATS2
AGPZ	0.17	258	72	6	4
BACO	0.26	272	127	6	completed
BAMO	0.32	1220	396	28	27
BENZ	0.30	716	258	9	completed
CF3BR	0.25	375	141	3	completed
CFCL	0.37	203	106	3	completed
CFI	0.37	429	149	3	completed
CIME	0.19	924	484	17	completed
CROX	0.21	657	202	15	completed
CUPZ	0.17	243	72	6	5
DADA	0.23	518	197	16	completed
LAMO	0.13	271	126	14	12
LASI	0.13	253	105	11	8
LEV	0.19	323	103	17	8
MES	0.20	719	229	13	11
METYL	0.27	318	169	5	completed
NBPO	0.25	1201	481	22	completed
NIZR	0.18	628	239	18	11
PBS	0.27	477	179	6	5
SAPO	0.17	717	183	21	9
SBPO	0.28	1071	337	17	13
SULPH	0.26	220	93	3	completed
YONO	0.27	680	203	18	completed

**Table 7:** For some test structures the phase error (the difference between the direct methods phases and the true phases) corresponding to the EXPO traditional Le Bail extraction run (ERR1) and to the use of the random procedure (ERR2) are given

Code	ERR1 (°)	ERR2 (°)
AND1	29.87	20.68
DADA	48.33	23.32
GAPO	37.18	29.90
LEV	73.55	32.53
UTM1	66.80	23.02
YURI	28.24	22.50

**Table 8:** For each test structure the number of feasible solutions (NFS), the best *R*_p_ value, the number of located anions (NA) respect to the true number (in parentheses), the average distance from the published atomic positions <*d*> and the CPU time are given

Code	NFS	*R*_p_	NA	<*d* > (Å)	CPU time^a^ (s)
CROX	5	0.22	11 (11)	0.24	33
EMT	2	0.12	12 (12)	0.23	103
MCM	3	0.23	15 (13)	0.23	257
NIZR	2	0.22	13 (12)	0.23	34
SAPO	6	0.29	10 (10)	0.27	38
SGT	6	0.49	11 (7)	0.45	98
UTM1	9	0.23	15 (13)	0.34	94
VFI	8	0.25	14 (14)	0.37	81
VNI	4	0.19	35 (30)	0.24	181

a Compaq^1^ personal Workstation 500au.

## Footnotes

1 Certain commercial equipment, instruments, or materials are identified in this paper to foster understanding. Such identification does not imply recommendation or endorsement by the National Institute of Standards and Technology, nor does it imply that the materials or equipment identified are necessarily the best available for the purpose.
